# A Direct Sulfation Process of a Marine Polysaccharide in Ionic Liquid

**DOI:** 10.1155/2015/508656

**Published:** 2015-05-19

**Authors:** Nathalie Chopin, Corinne Sinquin, Jacqueline Ratiskol, Agata Zykwinska, Pierre Weiss, Stéphane Cérantola, Jean Le Bideau, Sylvia Colliec-Jouault

**Affiliations:** ^1^IFREMER, Institut Français de Recherche pour l'Exploitation de la Mer, Laboratoire Ecosystèmes Microbiens et Molécules Marines pour les Biotechnologies, rue de l'île d'Yeu, BP 21105, 44311 Nantes Cedex 03, France; ^2^Institut des Matériaux Jean Rouxel (IMN), Université de Nantes, CNRS, 2 rue de la Houssinière, BP 32229, 44322 Nantes Cedex 3, France; ^3^INSERM U791, Laboratoire d'Ingénierie Ostéo-Articulaire et Dentaire (LIOAD), Université de Nantes, 1 place Alexis Ricordeau, BP 84215, 44042 Nantes Cedex 1, France; ^4^Laboratoire de Résonance Magnétique Nucléaire, Université de Bretagne Occidentale, CS 93837, 29238 Brest Cedex 3, France

## Abstract

GY785 is an exopolysaccharide produced by a mesophilic bacterial strain *Alteromonas infernus* discovered in the deep-sea hydrothermal vents. GY785 highly sulfated derivative (GY785 DRS) was previously demonstrated to be a promising molecule driving the efficient mesenchymal stem cell chondrogenesis for cartilage repair. This glycosaminoglycan- (GAG-) like compound was modified in a classical solvent (*N,N*′-dimethylformamide). However, the use of classical solvents limits the polysaccharide solubility and causes the backbone degradation. In the present study, a one-step efficient sulfation process devoid of side effects (e.g., polysaccharide depolymerization and/or degradation) was developed to produce GAG-like derivatives. The sulfation of GY785 derivative (GY785 DR) was carried out using ionic liquid as a reaction medium. The successful sulfation of this anionic and highly branched heteropolysaccharide performed in ionic liquid would facilitate the production of new molecules of high specificity for biological targets such as tissue engineering or regenerative medicine.

## 1. Introduction

**Figure 1:**
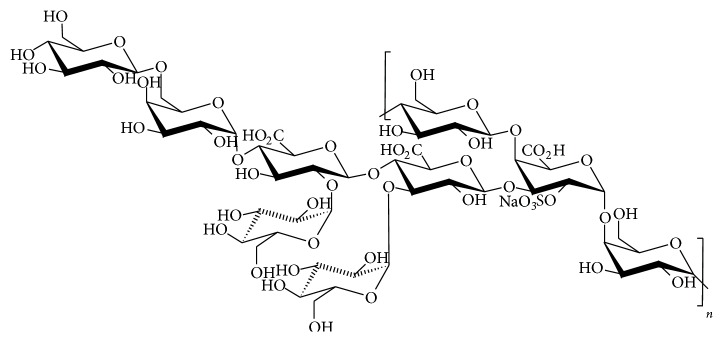
Structure of the monosulfated nonasaccharide repeating unit of the native bacterial GY785 EPS.

In cartilage tissue engineering, differentiation of mesenchymal stem cells into chondrocytes is crucial to obtain successful cartilage regeneration. The differentiation can be promoted by various biological agents, including polysaccharides. In peculiar, glycosaminoglycans (GAGs) were shown to participate in many biological processes including cell adhesion, migration, proliferation, and differentiation likely through interactions with proteins, such as growth factors, proteases, and chemokines [1, 2]. The presence of sulfate and carboxylic groups in GAG structure determines the interaction through electrostatic interactions [1]. For instance, heparin, which is a GAG widely known for its anticoagulant and antithrombotic properties [3], has been employed in the design of materials as engineered scaffolds for tissue regeneration and controlled release platforms for growth factor delivery [4]. However, undesirable side effects of this sulfated polysaccharide, such as hemorrhagic complications, heparin-induced thrombocytopenia, and low bioavailability [5] as well as its mammalian origin increasing the risk of the presence of infectious agents (e.g., viruses and prions), considerably limit its biological applications. In this context, marine microorganisms, such as marine bacteria, producing exopolysaccharides (EPS) with unique structures, become a promising source of new GAGs-like molecules with a low risk of contamination by pathogenic agents. Indeed, a mesophilic bacterial strain discovered in the deep-sea hydrothermal vents,* Alteromonas infernus*, was shown to excrete a water-soluble branched acidic heteropolysaccharide named GY785 (Figure 1) with a high molecular weight (HMW, i.e., >10^6^ g/mol) and low sulfur content (up to 3 wt%) [6]. This marine prokaryote was classified as a nonpathogenic microorganism by the Institute Pasteur (Paris, France). Moreover, the recent development of powerful bacterial engineering tools, such as large-scale fermenters, allows the production of the EPS at a viable economic cost by biotechnological processes [7].

The repeating unit of GY785 EPS consists of a monosulfated nonasaccharide composed of three uronic acids (two glucuronic acids and one galacturonic acid) and six neutral hexoses (four glucoses and two galactoses) [8]. The galacturonic acid unit is the only one sugar bearing a sulfate group at C2 position giving a slightly sulfated EPS (Figure 1). Structural modifications were shown to be essential for promoting the biological activity of this EPS and providing a GAG mimetic compound [9–12]. Firstly, a depolymerization step of the native HMW polysaccharide afforded a low-molecular weight (LMW) derivative, GY785 DR, and then a subsequent chemical sulfation reaction gave a highly sulfated LMW derivative, GY785 DRS, with a sulfur content up to 12 wt% [9]. Recently, the effect of this derivative on chondrogenesis was investigated and has shown that being added to a chondrogenic differentiation medium, containing chondrogenic growth factors such as TGF-*β*1, GY785 DRS enhanced the chondrogenic differentiation of mesenchymal stem cells, considered as an attractive source of cells for cartilage engineering [13].

The efficient production of semisynthetic GAG mimetic derivatives through chemical sulfation still remains a challenge since it presents several limitations: (i) bringing together hydrophilic natural macromolecules and organic reagents in homogeneous conditions, (ii) difficulties for driving the reaction to completion due to anionic interactions which increase with the number of linked sulfate groups, (iii) lack of control and reproducibility towards the reaction (e.g., regioselectivity), (iv) lability of sulfate groups in acidic conditions or at high temperatures, and (v) difficulty to remove high proportion of inorganic salts at the end of the reaction [14]. Considering that highly sulfated products have valuable biological targets and that the sulfate groups are sensitive, sulfation reaction is mostly performed as final chemical modification of the polysaccharide structure.

Sulfated polysaccharide derivatives can be obtained by a chemical modification under non homogeneous or homogeneous conditions. Homogeneous media are more attractive since they induce equal accessibility of polysaccharidic hydroxyl groups to the sulfating agent thus increasing the efficiency of sulfation. A wide range of studies describes classical solvent-systems, such as* N,N*′-dimethylacetamide (DMAc), lithium chloride (LiCl), dimethyl sulfoxide (DMSO), dimethylformamide (DMF), or tetrabutylammonium fluoride trihydrate (TBAF) to dissolve polysaccharides prior to their modification [15–18]. However, poor solubility of hydrophilic polysaccharides in organic solvents as well as important polysaccharide degradation during reaction limits their use as good solvents. Recently, ionic liquids (ILs) were shown to constitute innovative media for solubilization of polysaccharides thus promoting their efficient chemical modification [19]. Indeed, because ILs ease the dissolution of both organic and inorganic reagents, they are considered as excellent interfaces to promote carbohydrate chemistry [18–20]. The efficiency in disruption of the inter- and intramolecular hydrogen bonds in carbohydrates can be enhanced by using the hydrophilic IL, namely, 1-butyl-3-methylimidazolium chloride (BMImCl) [21–23]. Sulfation process in ILs has been mainly described for cellulose [24–27]. However, harsh reaction conditions used have caused an important backbone degradation [28–31].

In the present study, the IL mainly described as a new class of cellulose solvents was chosen for the first time to modify an anionic branched heteropolysaccharide, GY785 EPS. In order to develop a one-step and efficient sulfation process completely free of side effects (e.g., polysaccharide depolymerization and/or degradation) to produce GAG-like derivatives, the sulfation of GY785 DR in BMImCl medium was assessed. Moreover, in order to facilitate the transfer of the one-step process developed here into a large scale, only commercially available reagents were used.

## 2. Materials and Methods

### 2.1. Materials

Sulfur trioxide pyridine complex (SO_3_·Py, technical grade, 48.8–50.3% active SO_3_),* N,N*′-dimethylformamide (DMF, 99.8%, Extra Dry over Molecular Sieve, AcroSeal), and 4-dimethylaminopyridine (DMAP, 99%) were purchased from Acros Organics. 1-Butyl-3-methylimidazolium chloride (BMImCl, 98%, melting point, 72°C) was obtained from Solvionic. Spectra/Por 6 Prewetted Dialysis Tubing (MWCO 1,000 Da, 1.1 mL/cm) was obtained from Interchim. All the reagents were purchased with an analytical grade. All ultrafiltration was performed with Pellicon 2 system and a membrane cut-off at 1,000 Da (surface 0.1 m^2^ Cassette Hystream Novasep, Millipore).

### 2.2. Preparation and Purification of EPS and Its Derivatives

#### 2.2.1. Production of the Native GY785 EPS

GY785 EPS is produced by* Alteromonas infernus*, a deep-sea, aerobic, mesophilic, and heterotrophic bacterium isolated from a sample of fluid collected among a dense population of* Riftia pachyptila* in the vicinity of an active hydrothermal vent of the Southern depression of the Guaymas Basin (Gulf of California). The production and isolation of the EPS were previously described [32]. In the present study, GY785 EPS production was carried out at 25°C and pH 7.4 in a 30 L fermenter (Techfors 30 L INFORS, Switzerland). Briefly, 20 L of Zobell medium, a marine culture medium prepared with 5 g/L of bactopeptones, 1 g/L of yeast extract, and 35 g/L of sea salts, were introduced in the fermenter before addition of 2 L of cells suspension inoculum. The carbohydrate source necessary for biosynthesis of the EPS was 30 g/L of glucose added at the beginning of the batch. At the end of the fermentation process, the EPS was recovered from the growth medium by a centrifugation step followed by ultrafiltration and freeze-drying.

#### 2.2.2. Preparation of GY785 DR

GY785 DR was obtained by a free-radical depolymerization process. Briefly, 2.5 g of the native EPS was dissolved overnight in 350 mL of water in a water-jacketed glass vessel, without stirring. When a complete dissolution was achieved, an aqueous solution of the catalyst (copper(II)) was added under pH control and stirring. The resulting mixture was maintained at 60°C during the reaction and the pH was set at 7.5. A continuous addition of a diluted hydrogen peroxide solution (1 mL/min) was then started under controlled pH conditions using a pHstat (Hach and Lange). The reaction was stopped when the amount of peroxide solution to be added was reached. The polysaccharide chains were stabilized by an overnight room temperature reduction reaction with sodium borohydride. Excess of sodium borohydride was then quenched with an aqueous solution of acetic acid (10 M). The contaminating copper cations were chelated passing the previous aqueous solution through Chelex 20 resin (sodium form). The resulting solution was ultrafiltrated before being freeze-dried.

#### 2.2.3. Preparation of Highly Sulfated GY785 DRS

The sulfation of the GY785 DR was performed either in BMImCl or in DMF. Firstly, GY785 DR was sulfated in IL medium. Briefly, to a solution of premelted BMImCl (950 mg) at 90°C, GY785 DR (50 mg) was added in one portion in a Schlenk tube. For a complete dissolution, the resulting mixture was stirred for 4 h at 90°C under reduced pressure (100 mbar). DMAP (5 mg) was then added to the mixture, followed by a suspension of SO_3_·Py (250 mg) in DMF (0.5 mL). The reaction mixture was stirred for 2 h at 70°C under atmospheric pressure. After dilution with water (10 mL) and cooling to room temperature, the solution pH was adjusted to 7-8 with NaOH (3N). The final solution was dialyzed against distilled water for 72 h to remove BMImCl, DMAP, DMF, excess of SO_3_·Py, and potential degradation products before being freeze-dried.

The kinetic of sulfation was studied at 70°C by varying the time of sulfation: 0.5 h, 1 h, 2 h, and 4.5 h. The effect of temperature reaction was studied at four different temperatures: 65°C, 70°C, 90°C, and 120°C. Finally, the effect of sulfating agent was assessed by replacing the SO_3_·Py by the sulfur trioxide trimethylamine complex (SO_3_·Me_3_N) and sulfur trioxide* N,N*′-dimethylformamide complex (SO_3_·DMF).

As previously described, the sulfation of the GY785 DR in anhydrous DMF was preceded by a preparation of GY785 DR in a pyridinium salt form by cation exchange chromatography using Dowex HCR-S column (Dow Chemical) [10]. The eluent was immediately neutralized by addition of the pyridine (pH 7-8). GY785 DR (50 mg) recovered after freeze-drying of the GY785 DR pyridinium salt was solubilized in anhydrous DMF (100 mL) for 2 h at 45°C under continuous stirring. After a complete solubilization, SO_3_·Py (250 mg) was added to the mixture and sulfation was followed for 2 h at 45°C under stirring. After dilution with water (20 mL) and cooling to room temperature, the solution pH was adjusted to 7-8 with NaOH (3 N). The final solution was extensively dialyzed against distilled water for 72 h and freeze-dried.

#### 2.2.4. Purification of GY785 DRS

In order to obtain a homogeneous fraction of GY785 DRS with both molecular weight of 10,000 g/mol and low polydispersity, a predominant population of polysaccharide chains was selected by a gel filtration chromatography using an AKTA FPLC system (GE Healthcare Life Sciences) and refractometric detection (Gilson). The column XK 26/100 was filled with 500 mL Superdex 30 preparative grade gel (GE Healthcare Life Sciences). 5 mL of the sample, containing 400 to 700 mg of GY785 DRS, were injected by using a superloop and eluted with 0.1 M ammonium acetate for GYDRS sulfated with IL process and with deionized water for GYDRS sulfated with DMF process. Fractions of 10 mL were collected and pooled. GYDRS IL pool was additionally dialyzed against distilled water before being freeze-dried.

### 2.3. Characterization of GY785 Derivatives

#### 2.3.1. Molecular Weight and the Polydispersity Index Determination by HPSEC-MALS

The weight-average molecular weight (Mw), number-average molecular weight (Mn), and the polydispersity index (Ip = Mw/Mn) of the samples were determined by high-performance size exclusion chromatography (HPSEC) coupled with a multiangle light scattering detector (MALS, Dawn Heleos-II, Wyatt Technology) and a differential refractive index (RI) detector (Hitachi L2490). HPSEC system was composed of an HPLC system Prominence Shimadzu, a PL aquagel-OH mixed, 8 *μ*m (Varian) guard column (*U* 7.5 mm ×  *L* 50 mm), and a PL aquagel-OH mixed (Varian) separation column (*U* 7.5 × 300 mm, operating range 10^2^–10^7^ g/mol). GY785 DR and GY785 DRS were dissolved in distilled water at a concentration of 2 mg/mL and filtered through 0.45 *μ*m cellulose acetate syringe filter before being injected. The elution was performed at 1 mL/min rate with 0.1 M ammonium acetate containing 0.03% NaN_3,_ filtered through 0.1 *μ*m membrane (Durapore Membrane, PVDF, Hydrophilic type VVLP, Millipore). Data were computed with Astra software 6.1 (Wyatt Technology).

#### 2.3.2. Determination of the Linked Sulfate Groups by HPAEC

The linked ester sulfate group content in the GY785 samples was determined using high-performance anion-exchange chromatography (HPAEC) by calculating the difference between the total sulfur contents present in the hydrolyzed sample and in the nontreated sample (free sulfur). Briefly, an aqueous solution of an internal standard, KNO_3_ (10 g/L), was added to an aqueous solution of GY785 DRS (2 mg/mL). The mixture was hydrolyzed with HCl (1 M) at 133°C for 4 h20. After cooling to room temperature and the addition of water (4.5 mL), 800 *μ*L of the sample was analyzed by HPAEC (in triplicate). Before injection, all samples were filtered through membrane filters with 0.45 *μ*m pore size. The sulfate peak was attributed with retention time reference to Na_2_SO_4_ standard, investigated over the concentration range 1–8 mM/L. The peak area response was found to be linear over this range (*r* ^2^ ≥ 0.997). HPAEC analyses were carried out with Dionex DX-500 ion chromatographic instrument controlled using Chromeleon software (version 6.80). The chromatographic system was composed of Dionex GP40 gradient pump, EG40 eluent generator with EGC II KOH eluent generator cartridge (EluGen II Hydroxide), ED 40 electrochemical detector with ASRS 300 4 mm conductivity suppressor, AS40 autosampler, and column compartment Ultimate 3000. Sample separation was conducted with the use of Dionex IonPac AS11-HC (250 mm × 4 mm) analytical anion exchange column with a guard column IonPac AG11-HC (50 mm × 4 mm).

#### 2.3.3. Elemental Analysis

The total sulfate content was also determined by the elemental analysis performed by the Central Microanalysis Department of the CNRS (Gif-sur-Yvette, France).

#### 2.3.4. ATR-FTIR and NMR Spectroscopy

Infrared spectra of GY785 DR and GY785 DRS were recorded with a FT-IR VERTEX 70 spectrometer (Bruker) in ATR mode in the range 4000–500 cm^−1^.

NMR ^1^H and ^13^C spectra were recorded using Bruker Avance 500 spectrometer (Bruker BioSpin, Wissembourg, France) equipped with a 5 mm ^1^H/^13^C/^15^N TCI cryoprobe at 25°C. GY785 DR and GY785 DRS were dissolved in 700 *μ*L of 99.96% deuterium oxide. Chemical shifts were expressed in parts per million (ppm) relative to tetramethylsilane (TMS) used as a reference.

## 3. Results and Discussion

*Alteromonas infernus* produces a water-soluble highly branched EPS, GY785, of high molecular weight (>10^6^ g/mol) and low sulfur content (up to 3 wt%) [6]. In order to improve the biological properties of the native EPS and to obtain a GAG-like compound, the radical depolymerization leading to a low molecular weight GY785 DR followed by its chemical sulfation was shown to be necessary. Indeed, only the GY785 DRS with a sulfur content up to 12% was shown to stimulate the chondrogenic differentiation of the mesenchymal stem cells most likely through interaction with the growth factor, TGF-*β*1 [13]. To get further insight into the regenerative mechanism induced by GY785 DRS, LMW sulfated polysaccharides with well-defined structural features (i.e., a molecular weight and a sulfur content) are required. In this context, a new method of polysaccharide sulfation was developed using IL as a reaction medium. In fact, the use of IL as solvent allows the reaction to be carried out in a homogeneous medium in one-step process, whereas, in classical solvents, such as DMF, an additional ion-exchange step leading to the polysaccharide in a pyridinium salt form is necessary to improve its solubility prior to sulfation [33]. GY785 DR sulfation in IL medium should lead to the sulfur content close to 12 wt%, which confers to the resulting GY785 DRS its biological activity, as previously assessed [13].

### 3.1. Sulfation of GY785 DR

The sulfation reaction of GY785 DR in BMImCl was performed in one-step process without converting the polysaccharide into a pyridinium salt form. In order to enhance the nucleophilicity of the hydroxyl groups on the carbohydrate backbone, the efficient sulfating agent, namely, sulfur trioxide pyridine complex (SO_3_·Py) [34], was chosen. In addition, the catalyst, 4-dimethylaminopyridine (DMAP), was added to the reaction mixture to increase the reaction rate thus decreasing the undesired depolymerization of the polysaccharide [28]. A small quantity of DMF was also added during the sulfation reaction to reduce the viscosity of the reaction medium. In parallel, the sulfation of GY785 DR was carried out in classical solvent, DMF [10].

A significant increase in both molecular weight (11,000 g/mol) and sulfur content (10.4 wt%) was observed for GY785 DRS obtained after sulfation performed in BMImCl, in comparison to GY785 DR before sulfation (7,500 g/mol and 3 wt% S). Indeed, a 1.5-fold increase in molecular weight was measured after sulfation, whereas the sulfur content rose 3.3 times (Table 1). The sulfur content obtained after sulfation in BMImCl was close to 10 wt%, as in DMF, giving a GY785 DRS with the capability to stimulate the chondrogenic differentiation of the mesenchymal stem cells [13].

A slight difference between the sulfur content determined by the elemental analysis and by HPAEC was observed and was partly due to the fact that the elemental analysis assesses the total sulfur content. In contrast, HPAEC allows us to determine the sulfur content directly linked to the polysaccharide by measuring the difference between the free sulfur present in the hydrolyzed and not hydrolyzed sample. In addition, a good sulfation reproducibility of GY785 DR with the sulfur content varying from 9 wt% to 11.6 wt% and a systematic increase in the molecular weight (data not shown) demonstrate that the sulfation reaction performed in BMImCl allows the efficient polysaccharide modification devoid of side effects (e.g., depolymerization or degradation).

**Figure 2:**
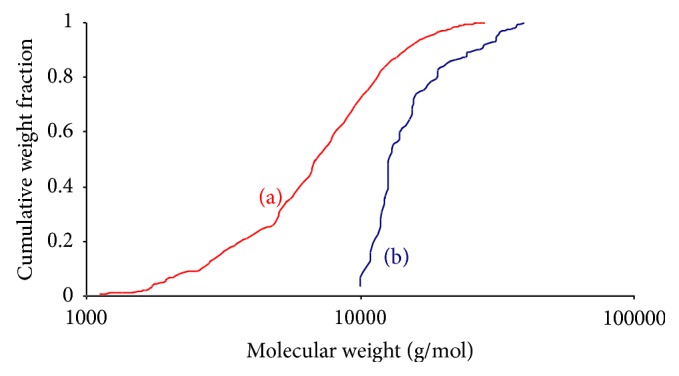
Cumulative molecular weight fraction of GY785 DRS with the dissolution step performed under atmospheric pressure (a) or under vacuum (100 mbar) (b).

It should be noted that the solubilization step preceding the sulfation reaction needs to be carried out under reduced pressure (100 mbar), since an important chain degradation was observed during dissolution under atmospheric pressure (Figure 2). Indeed, at atmospheric pressure, a heterogeneous molecular weight distribution was obtained, with chain molecular weights ranging from 300 to 10,000 g/mol, whereas, a narrow molecular weight distribution was observed when the dissolution was performed under reduced pressure, most of the chains having a molecular weight close to 10,000 g/mol (Figure 2). The observed degradation of the polysaccharide backbone under atmospheric pressure is most likely due to the presence of residual amount of water since GY785 DR and BMImCl are both hygroscopic compounds. Therefore, the water content must be reduced to permit firstly an easy dissolution of the polysaccharide in the IL and then to avoid the hydrolysis of the EPS by sulfuric acid formed when the sulfating agent SO_3_·Py is added to the reaction medium [35]. To limit the moisture content and to prevent the polysaccharide degradation and/or depolymerization, the dissolution step was performed for 4 h at 90°C, that is, a temperature higher than the melting point of the IL, and under reduced pressure (100 mbar).

The new sulfation process of GY785 DR developed in BMImCl as the reaction medium was compared to the classical sulfation method involving DMF as solvent [10]. The sulfation of GY785 DR in DMF led also to an increase in both molecular weight (16,000 g/mol) and sulfur content (16 wt%), when compared to GY785 DR (Table 1). Slightly higher sulfur content obtained after sulfation in DMF, when compared to sulfation proceeded in BMImCl (10 wt%), was most likely due to the fact that GY785 DR was firstly converted into its pyridinium salt before sulfation, which enhances its solubility and improves its modification [10]. However, higher sulfur content obtained in DMF compared to IL medium could not be necessarily beneficial. With a high degree of GAG sulfation, an increase in transient growth factor sequestration is observed and the intensity of growth factor activity (enhanced or inhibited) can be manipulated. Consequently, to regulate properly the sequestration (that is, the enhancement of growth factor activity but not its inhibition, which could occur with a too strong interaction between GAG and growth factor), a sulfur content close to 10 wt% is required for bioinspired GAG design [36].

### 3.2. Analyses of LMW-S Derivatives

**Figure 3:**
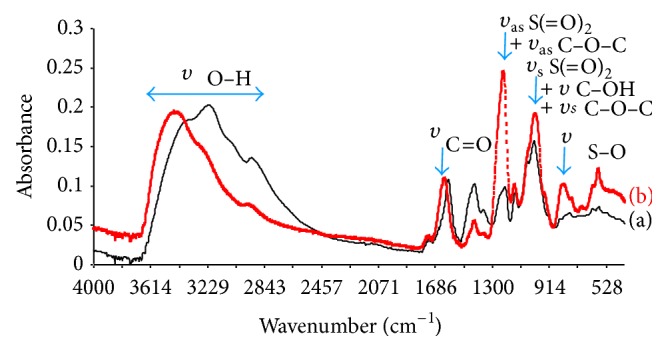
ATR FT-IR spectra of GY785 DR (a) and GY785 DRS sulfated in BMImCl medium (b).

ATR FT-IR spectra of initial GY785 DR and GY785 DRS obtained after sulfation in BMImCl medium are presented on Figure 3. Both spectra exhibited a broad O–H stretching band at 3600–3000 cm^−1^, a minor C–H stretching band at 2900 cm^−1^, a typical C=O stretching band at 1650 cm^−1^, and a strong absorption band at 1050 cm^−1^ assigned to the C–O and C–O–H vibrations. However, the differences between the two spectra were noticeable. The band associated with O–H was considerably reduced in GY785 DRS due to the esterification of the hydroxyl groups during sulfation reaction. In addition, strong bands at 1250 cm^−1^ and 816 cm^−1^, observed in GY785 DRS spectrum, assigned, respectively, to S=O asymmetric stretching and S–O symmetric vibrations, indicate clearly that the sulfation of hydroxyl groups actually occurred.

**Figure 4:**
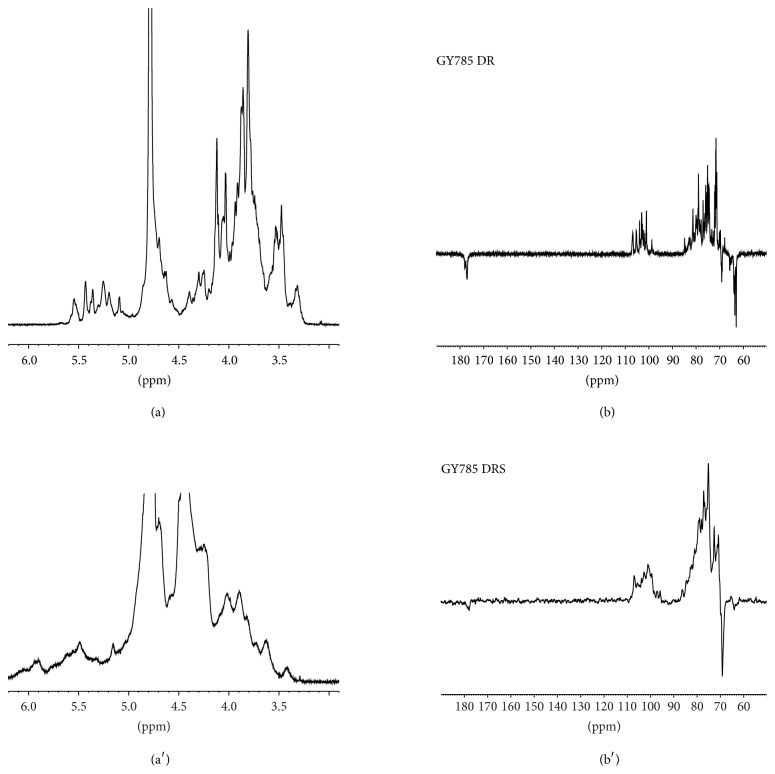
^1^H NMR spectra of GY785 DR (a) and GY785 DRS (a′) and ^13^C J-modulated NMR spectra of GY785 DR (b) and GY785 DRS (b′).

The efficiency of sulfation was also demonstrated by NMR spectroscopy. ^1^H and ^13^C NMR spectra of GY785 DR and GY785 DRS are presented on Figure 4. In ^1^H NMR spectra, peaks corresponding to anomeric region became complex after sulfation reaction and a large peak at 4.4 ppm and new signals at 6 ppm appeared. More complex ^13^C NMR spectrum of GY785 DRS and the CH_2_ downfield chemical shift observed at 70 ppm indicated the substitution of CH_2_–OH groups after sulfation reaction. However, further extensive NMR analyses have to be carried out in order to determine precisely the sulfation pattern and the position of added sulfate groups on the GY785 DRS backbone [37].

In order to get further insight into the sulfation of GY785 DR in BMImCl, the influence of time, temperature, and sulfation agent on both molecular weight and sulfur content was studied.

### 3.3. Effect of the Reaction Time on the Sulfation Reaction

**Figure 5:**
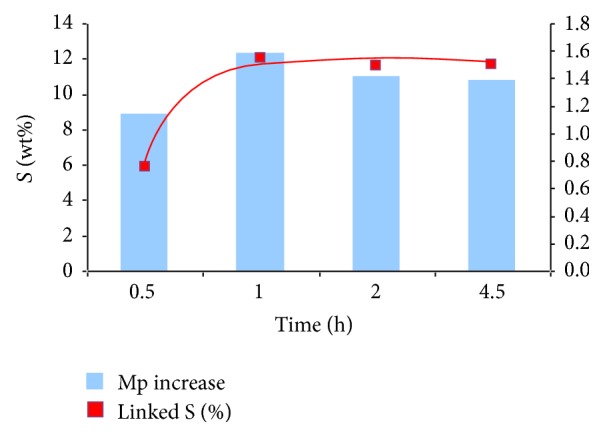
Comparison of the variation of the molecular weight and the sulfur content in GY785 DRS recovered following different sulfation time.

Firstly, the kinetic of sulfation in BMImCl was studied. For this purpose, the sulfation was performed for four different reaction times: 0.5 h, 1 h, 2 h, and 4.5 h.  Figure 5 presents, for each reaction time, the variation of the molecular weight before and after sulfation (blue bar giving the multiplier between the two molecular masses), while the red line represents the sulfur content (wt%) in the final GY785 DRS. The rapid increase in the sulfur content in GY785 DRS suggests that the esterification of the hydroxyl groups was fast and occurred in the first minutes of the reaction to reach at 1 h a level of 12 wt% of the sulfur content. The efficiency of the esterification reaction could be explained by the presence of DMAP catalyst which promoted and accelerated the substitution step. DMF added during the sulfation reaction reduced the solution viscosity, which improved homogeneous sulfation conditions. When the reaction time was increased above 1 h, no further increase in the sulfur content was observed (11 wt%). However, a slight decrease in the variation of molecular weight was noticed with increasing reaction time, which was most likely due to the unwanted polymer backbone degradation initiated by hydrolysis by sulfuric acid formed from the sulfating agent [35].

### 3.4. Effect of Temperature on the Sulfation Reaction

In order to assess the effect of temperature on sulfation efficiency, the reaction was carried out at four different temperatures: 65°C, 70°C, 90°C, and 120°C (Table 2). Similar results were obtained when sulfation of GY785 DR was performed at the temperatures between 70°C and 120°C. Indeed, the sulfur content in GY785 DRS varied only slightly between 10 wt% and 11 wt%. At 120°C, even if a satisfying sulfur amount of 10 wt% was obtained, a brown aspect of the freeze-dried final product and higher polydispersity index suggests the degradation of the polysaccharide structure. The sulfation performed at 65°C led to a lower sulfur content, as only 7.6 wt% was measured. It becomes therefore that a temperature between 70°C and 90°C is more suitable for the sulfation reaction of GY785 DR in BMImCl.

### 3.5. Effects of Sulfation Agent on the Sulfation Reaction

In order to assess the influence of the nature of sulfating reagent, in addition to SO_3_·Py, two commercially available reagents were selected: sulfur trioxide trimethylamine complex (SO_3_·Me_3_N) and sulfur trioxide* N,N*′-dimethylformamide complex (SO_3_·DMF). The well-known sulfur trioxide complex associated with aromatic amine pyridine (SO_3_·Py) used in the present study to develop a sulfation process in IL was used as a reference. The second sulfation reagent was SO_3_·Me_3_N complex, where trimethylamine is a stronger base than pyridine. The tertiary amine released from the complex during the reaction plays a role of acid scavenger [38] and should prevent the polysaccharide from acidic degradation. The third sulfation agent selected, SO_3_·DMF complex, shows different assets. DMF is a weaker base than pyridine, which implies that the partial positive charge on the sulfur atom of the SO_3_·DMF complex would be greater than that in the SO_3_·Py complex. Thus, the nucleophilic attack on the SO_3_·DMF complex would be promoted and could increase the sulfur amount in the final product. Moreover, during the sulfation reaction DMF could also be released from the sulfating complex, which may decrease the medium viscosity and enhance sulfation as well.

Similar sulfur content obtained after sulfation with different sulfating agents (varying from 10.4 wt% to 10.7 wt%, Table 3) suggests that the efficiency of sulfation is similar whatever the sulfating agent used. This could be explained by important structural complexity of GY785 DR, which results in low accessibility of hydroxyl groups for sulfation reaction. In addition, SO_3_·DMF, more reactive sulfation complex than SO_3_·Py, could be more rapidly degraded by residual water present in the reaction medium, which may decrease its efficiency as a sulfating agent.

More differences were observed in the molecular weights after sulfation. Indeed, the sulfation procedure using SO_3_·Py complex afforded a polymer chains with the highest molecular weight (10,000 g/mol) and a narrow polydispersity. On the contrary, for the same sulfur content, an increase in molecular weight of GY785 DRS sulfated with SO_3_·Me_3_N or SO_3_·DMF was lower (9,000 g/mol) when compared to GY785 DRS sulfated with SO_3_·Py. In addition, the polydispersity index slightly increased after sulfation (Table 3). Lower molecular weights and higher polydispersity indexes of GY785 DRS sulfated with SO_3_·Me_3_N or SO_3_·DMF suggest that the polysaccharide structure was slightly degraded during the reaction. Therefore, SO_3_·Py remains as the best sulfating agent for GY785 DR modification.

## 4. Conclusions

In the present work, a one-step chemical sulfation process was successfully developed using BMImCl as a reaction medium without undesired degradation of the polysaccharide backbone. The homogeneous dissolution of the polysaccharides in IL was the main challenge of this process. The sulfation reaction was shown to be optimal at 70°C using SO_3_·Py/DMAP/DMF as the sulfation system. The final GY785 DRS was obtained with a good yield and the reproducibility of sulfation by a rigorous control of both sulfur content and molecular weight was assessed. Moreover, it was demonstrated that the sulfur content can be modulated by varying the temperature or the time of the reaction. In conclusion, the new sulfation process developed in the present study using IL as reaction medium appears as a promising method leading to new GAG mimetic derivatives from marine origin. Further studies of GY785 DRS fine structure (i.e., the position of the sulfate groups on the polysaccharide backbone) would help to understand the influence of the structure on the biological properties of GY785 DRS.

The production of tailor-made oligosaccharide or polysaccharide structures by biotechnological process is a growing field of interest. This kind of process would facilitate the production of new macromolecules with high specificity for biological targets such as tissue engineering or regenerative medicine.

## Figures and Tables

**Table 1:** Molecular weight, Mw (g/mol), the polydispersity index, Ip and sulfur content, S (wt%) of GY785 DR and GY785 DRS (before and after sulfation, respectively).

Derivative	Solvent	Temp. (°C)	$\overset{-}{\text{Mw}}$ (g/mol)	Ip	S^*^ (wt%)	S^**^ (wt%)
GY785 DR	—	—	7,500	1.1	3.4	3.0

GY785 DRS	BMImCl	70	11,000	1.1	9.7	10.4
	DMF	45	16,000	1.1	16.4	16.0

^*^Determined by the elemental analysis.

^**^Determined by HPAEC method.

**Table 2:** Molecular weight, Mw (g/mol), the polydispersity index, Ip and sulfur content, S (wt%) of GY785 DR and GY785 DRS obtained after sulfation carried out at different temperatures.

Derivative	Temp. (°C)	$\overset{-}{\text{Mw}}$ (g/mol)	Ip	S^*^ (wt%)	S^**^ (wt%)
GY785 DR	—	7,500	1.1	3.4	3.0

GY785 DRS	65	9,000	1.1	8.2	7.6
	70	11,000	1.1	9.7	10.4
	90	10,500	1.1	11.2	11.1
	120	8,000	1.3	10.7	10.1

^*^Determined by the elemental analysis.

^**^Determined by HPAEC method.

**Table 3:** Molecular weight, Mw (g/mol), the polydispersity index, Ip and sulfur content, S (wt%) of GY785 DR and GY785 DRS obtained after sulfation with different sulfation agents.

Derivative	Sulfation agent	Mw (g/mol)	Ip	S^*^ (wt%)	S^**^ (wt%)
GY785 DR	—	7,500	1.1	3.4	3.0

GY785 DRS	SO_3_·Py	10,000	1.1	9.7	10.4
	SO_3_·DMF	9,000	1.3	11.7	10.5
	SO_3_·Me_3_N	9,000	1.2	7.5	10.7

^*^Determined by the elemental analysis.

^**^Determined by HPAEC method.
